# The Problem of the Midwife

**Published:** 1917-07-14

**Authors:** 


### BABY WEEK CONFERENCES AT WESTMINSTER.

### The Problem of the Midwife.

It is matter for satisfaction that the meetings of this Conference were, with the curious exception of that upon children under the Poor Law, so well attended. It must be confessed that the problem of the midwife's work, especially in rural districts, grows in complexity under discussion. It occupied the first day (Wednesday), and was supplemented by a meeting of the Society for the Training of Midwives at Lady Glenconner's house on Friday, July 6. Sir Robert Morant, who took the chair on the afternoon of the 4th, laid stress on the idea of an itinerant midwife who might solve the difficult question of headquarters by living with one working within the area of a local association, and travel round outlying farms. Miss Burnside's array of facts as to the difficulties were impressive enough; capable women are not likely to join a profession in which they learn that a skilled woman was only able to support herself adequately by taking in children to nurse, and that only because she had a mother who cared for them in her absence. Yet if district nursing in her county of Herts were put upon the rates, a 1d. rate would suffice. Dr. Janet Lane Claypon urged a 25s. fee, and laid stress, as did other speakers, themselves practical midwives, on the need that inspectors should be competent and experienced, and not merely persons of good education who had added the C.M.B. diploma to their outfit in order to take such employment. In one case the nurse had been threatened with inspection by one of her own pupils. A plea was put in for the service of the "handy woman" to help the skilled midwife as she now helps the doctor. It would seem that the agricultural country of Denmark has tolerably well solved the problem. There the midwife pays about £2 10s. for training in Copenhagen after a month's probation. She then lives where she desires under licence from a magistrate, and under condition of calling in a doctor to abnormal cases. She receives from Government a retaining fee of £8 or £9, with eventual pension, plus a cottage of four or five rooms and grazing for a cow. Patients pay 6s. to 30s. She is liable to a fine or deprivation of licence for misconduct.

